# A deterministic large language model (LLM) framework for safe, protocol-adherent clinical decision support: application in hemodialysis anemia management (AnemiaCare HDs)

**DOI:** 10.3389/frai.2025.1728320

**Published:** 2025-12-12

**Authors:** Jose Arriola-Montenegro, Charat Thongprayoon, Benjamin Bizer, Jing Miao, Karina Ordaya-Gonzales, Iasmina M. Craici, Wisit Cheungpasitporn

**Affiliations:** Department of Internal Medicine, Division of Nephrology and Hypertension, Mayo Clinic, Rochester, MN, United States

**Keywords:** anemia management, artificial intelligence, end-stage kidney disease, hemodialysis, large language models

## Abstract

**Background:**

Large language models (LLMs) show promise for clinical decision support but often deviate from evidence-based protocols, raising safety and regulatory concerns. Anemia management in hemodialysis patients requires strict adherence to erythropoiesis-stimulating agent (ESA) and intravenous (IV) iron dosing rules, making it a high-risk use case for uncontrolled model behavior. To address this gap, we developed AnemiaCare HD, a deterministic LLM framework engineered to deliver transparent, reproducible, and protocol-adherent clinical recommendations.

**Methods:**

AnemiaCare HD was evaluated using 600 simulated hemodialysis anemia scenarios derived from a standardized institutional protocol. The model required six fixed clinical inputs (hemoglobin, hemoglobin rate of change, trend direction, transferrin saturation, ferritin, and current ESA dose). Phase 1 tested a loosely structured prompt. Phase 2 implemented deterministic prompt logic incorporating ESA kinetics, iron dosing rules, mandatory timing safeguards, and embedded safety alerts. Two independent nephrologists assessed protocol adherence.

**Results:**

In Phase 1, only 96 of 300 cases (32%) aligned with protocol recommendations, with common errors in ESA titration, iron dosing, and timing violations. In Phase 1, loosely structured prompting produced variable outputs, with only 96 of 300 simulated cases (32%) fully protocol-adherent and frequent unsafe recommendations. In contrast, deterministic prompting in Phase 2 resulted in 100% adherence across all 300 cases, eliminating protocol deviations, unsafe iron dosing, and timing violations (*p* < 0.001). In Phase 2, deterministic encoding achieved full protocol adherence (300/300, 100%), eliminating unsafe or premature recommendations (*p* < 0.001 vs. Phase 1) and consistently generating structured, rationale-based outputs.

**Conclusion:**

Deterministic LLM engineering enables safe, fully protocol-compliant clinical decision support in high-risk therapeutic domains. AnemiaCare HD demonstrates the viability of regulatory-aligned, auditable LLM frameworks for clinical use, although real-world integration and prospective validation remain necessary next steps.

## Introduction

Anemia is among the most common and clinically significant complications of end-stage kidney disease (ESKD). With the global burden of ESKD projected to rise to nearly six million people by 2030, anemia represents a major challenge, affecting roughly half of these patients (Liyanage et al., 2015; Kovesdy et al., 2023). Among those receiving hemodialysis, anemia most often develops because of reduced erythropoietin (EPO) production, disruptions in iron metabolism associated with chronic inflammation, blood loss, and oxidative stress (Fishbane and Spinowitz, 2018; Astor et al., 2002; Ifudu et al., 1996; Escandell-Montero et al., 2014; Babitt and Lin, 2012; Ku et al., 2023). Hemoglobin (Hb), the key protein responsible for oxygen transport, depends on both EPO stimulation of red blood cell precursors and adequate iron availability for its synthesis (Fishbane and Spinowitz, 2018; Cavill, 2002). When these mechanisms are impaired, Hb levels decline, leading to fatigue, diminished quality of life, higher cardiovascular risk, and increased mortality (Fishbane and Spinowitz, 2018; Bárány et al., 1993; Foley et al., 1996; Lefebvre et al., 2006; Locatelli et al., 2004).

The introduction of recombinant human EPO transformed anemia management by reducing the need for transfusions and improving patient outcomes (Barbieri et al., 2016a; Bazeley and Wish, 2019; Ifudu et al., 1995; Drüeke and Parfrey, 2012; Анемії, 2012). Equally important, the use of intravenous (IV) iron has become a cornerstone of therapy, as ongoing iron supplementation is critical to sustain erythropoiesis and optimize the effectiveness of ESAs (Drüeke and Parfrey, 2012; Анемії, 2012; Anumas et al., 2023; Macdougall et al., 2019; Babitt et al., 2021). Together, these therapies provide the foundation of anemia care in hemodialysis. However, despite their central role, achieving the correct balance between ESAs and IV iron remains complex and time-consuming for clinicians (Ifudu et al., 1995; Anumas et al., 2023; Besarab et al., 2000; Coyne, 2006; Charytan et al., 2015; Besarab, 2006; Hung and Tarng, 2014). Hb levels in dialysis patients frequently fluctuate above and below the target range, a phenomenon known as Hb cycling (Jörg et al., 2023; Collins et al., 2005; van der Putten et al., 2009; Thanakitcharu and Jirajan, 2016; Plappert et al., 2024). This instability often arises when ESA doses are adjusted too frequently or applied too rigidly, without accounting for patient variability or the delayed pharmacodynamic effect of each dose (Escandell-Montero et al., 2014). As a result, Hb levels swing between under- and over-correction, creating cycles that are difficult to stabilize in routine practice. Hb cycling has been linked to adverse outcomes and substantially increases the cost of anemia management, underscoring the need for safer and more consistent treatment strategies (Fishbane and Berns, 2007; Collins et al., 2012; Boudville et al., 2009; Swaminathan et al., 2015).

Machine learning models have been explored to improve dosing precision (Escandell-Montero et al., 2014; Barbieri et al., 2016a,b; Gaweda et al., 2008; Tuck et al., 2017; Barbieri et al., 2015; Kang et al., 2024; Ohara et al., 2021; Yun et al., 2021; Yang et al., 2023), but most focus narrowly on ESA adjustment, lack interpretability, or fail to ensure full compliance with established protocols (Barbieri et al., 2015, 2016a). However, while several prior AI and machine-learning approaches have incorporated both ESA and iron management, including the Anemia Control Model (ACM) (Garbelli et al., 2024a,b; Gandjour et al., 2025), which integrates iron dosing algorithms and has been deployed internationally, the majority of these systems emphasize dose optimization and predictive control rather than transparent, rule-based protocol enforcement. Existing models often function as black-box optimizers, providing limited visibility into the rationale behind dosing decisions and offering variable adherence to institution-specific safety constraints. In contrast, the present framework was designed to ensure complete protocol fidelity through deterministic rule encoding and explicit safety guardrails while maintaining clinician-facing natural-language justification. Recently, large language models (LLMs) have emerged as adaptable clinical reasoning tools; however, general-purpose LLMs are prone to hallucinations, inconsistent recommendations, and unsafe deviations from dosing thresholds, limiting their suitability in high-risk therapeutic domains such as dialysis anemia management (Ohara et al., 2021; Yun et al., 2021; Yang et al., 2023).

To address this gap, we developed AnemiaCare HD, a deterministic LLM framework engineered to deliver safe, reproducible, and protocol-adherent anemia management recommendations for hemodialysis patients. The system incorporates explicit ESA and IV iron dosing rules, pharmacodynamic timing safeguards, and integrated safety checks to ensure full alignment with institutional protocols. We evaluated its performance across 600 simulated anemia scenarios derived from a standardized protocol. This work demonstrates, for the first time, that deterministic prompt design can achieve complete fidelity to a complex clinical protocol in a high-risk therapeutic domain, overcoming a major barrier to safe deployment of LLM-based clinical decision support.

## Methods

### Study design and setting

AnemiaCare HD was evaluated in two phases using 600 simulated anemia management cases. All cases were based on institutional anemia protocols and designed to represent a wide range of scenarios, including both common and edge cases (e.g., Hb < 9 g/dL, Hb > 12.5 g/dL, iron deficiency, and ferritin >1,200 ng/mL). No real patient data were used, ensuring the study remained entirely simulation-based. All protocol rules applied in the simulation were directly referenced to the institutional anemia management algorithm used in clinical practice, including ESA titration increments, timing safeguards, and dual-parameter iron dosing criteria. The full rule set is provided in Table 1 to support transparency and reproducibility.

**Table 1:** Institutional anemia protocol rules encoded into AnemiaCare HD.

Clinical scenario	Protocol rule applied	Model action (deterministic response)	Safety note triggered
Hb < 9 g/dL with slow decline	Increase ESA by +10 mcg	Generates ESA ↑ (+10 mcg)	Monitor for rapid drop
Hb > 12.5 g/dL	Hold ESA	Output: discontinue ESA—review with nephrology	“Hb > 12.5 g/dL → hold ESA”
TSAT < 20%, ferritin < 200 ng/mL	IV iron loading 200 mg × 5	Output: initiate loading protocol	“Reassess ferritin post-course”
TSAT 20–29%, ferritin 200–800 ng/mL	100 mg IV iron weekly	Output: continue maintenance dose	“Stop if ferritin > 800 ng/mL”
Ferritin > 1,200 ng/mL	Stop IV iron	Output: discontinue iron immediately	“Iron overload risk”
Hb rise > 1.0 g/dL in 2 weeks	ESA hold × 2 weeks → restart −25% dose	Output: hold ESA; resume reduced dose	“Rapid Hb rise alert”

ESA, rythropoiesis-stimulating agent; ferritin, Iron storage protein concentration in serum (ng/mL); Hb, hemoglobin (g/dL); IV, intravenous; mg, milligrams; ng/mL, nanograms per milliliter; TSAT, transferrin saturation (%). This table summarizes the institutional anemia protocol example rules encoded into AnemiaCare HD. Each rule includes the clinical trigger, the deterministic model response, and the safety note generated. These ensure full transparency, reproducibility, and auditability.

To construct the 600 simulated scenarios, we used a stratified sampling approach designed to cover the full decision space of the institutional protocol rather than relying on random generation. Hemoglobin values were sampled across predefined strata (5.0–8.9, 9.0–10.7, 10.8–12.5, and >12.5 g/dL), with balanced representation of increasing and decreasing trends and varying rates of change (−2.0 to +2.0 g/dL/week). Iron indices were similarly stratified across TSAT <20%, 20–29%, 30–35, and >35% and ferritin <200, 200–800, 801–1,200, and >1,200 ng/mL categories. ESA doses ranged from 0 to 200 mcg/week, reflecting clinically observed dosing distributions. Approximately one-third of cases incorporated edge or conflict scenarios that challenge protocol boundaries (e.g., ferritin >1,200 ng/mL with TSAT <15%, rapid Hb rise despite low ESA dose), ensuring evaluation under clinically difficult conditions. This structured sampling strategy prevented overrepresentation of straightforward cases and enabled stress testing of protocol logic and deterministic rule execution.

In addition, edge and conflict scenarios were intentionally oversampled to ensure robust evaluation of safety mechanisms. Approximately one-third of all simulated cases involved protocol boundary conditions or discordant indices, such as Hb > 12.5 g/dL, ferritin >1,200 ng/mL, TSAT <15% with markedly elevated ferritin, or rapid Hb increases despite low ESA dosing. These high-risk combinations were selected because they require activation of protocol safety rules, including ESA holds, iron discontinuation, and clinician review. Their inclusion allowed systematic assessment of whether deterministic rule encoding and output constraints reliably prevented unsafe recommendations under challenging clinical conditions.

- Phase 1: baseline testing with a loosely defined natural language prompt.
- Phase 2: testing with a fully deterministic prompt incorporating explicit ESA and iron rules, timing safeguards, and safety checks.

### Model architecture

AnemiaCare HD was developed on a GPT-based framework that was deliberately constrained to behave deterministically. To support reproducibility, the deterministic version of AnemiaCare HD was executed using a fixed model configuration that included documented model versioning, zero-temperature sampling (temperature = 0.0), disabled nucleus sampling (top-*p* = 1.0), and a fully constrained output template. The system prompt and input sequence were identical across all 300 Phase II cases, and output generation occurred within a single platform release. All prompts, model settings, and outputs were archived to enable independent verification. In addition, the model required six mandatory clinical inputs provided in a fixed order, and no recommendations were generated if any input was missing or ambiguous, ensuring deterministic behavior. To guarantee reproducibility, the model operated within an 8,000-character limit and required six mandatory clinical inputs, collected in strict sequence:

1. Hb (g/dL)
2. Rate of Hb change (g/dL/week, over 2–4 weeks)
3. Direction of Hb trend (increasing or decreasing)
4. Transferrin saturation (TSAT, %)
5. Ferritin (ng/mL)
6. Current weekly Aranesp dose (mcg)

The model did not generate recommendations unless all six values were provided, eliminating errors from incomplete or ambiguous data.

Output generation was constrained through a fixed, rule-based template that required the model to populate predefined fields, including ESA recommendation, iron therapy status, timing interval, and safety notes. Free-text generation outside these fields was blocked, and each clinical trigger could map only to a limited set of allowable outputs defined by the institutional protocol (e.g., “increase ESA by +10 mcg,” “hold ESA,” “discontinue iron”). If the model attempted to produce text outside the permitted structure or suggest a dose or timing change not supported by protocol logic, the system defaulted to a standardized safety message requesting clinician review. This template-based constraint ensured consistency, prevented uncontrolled generative outputs, and operationalized deterministic behavior across all Phase II cases.

Each input variable was required to fall within predefined physiologic and protocol-based ranges (e.g., Hb 5–15 g/dL, TSAT 0–60%, ferritin 50–2000 ng/mL, rate of Hb change −2.0 to +2.0 g/dL/week, and ESA dose 0–200 mcg/week). The model verified completeness and plausibility of all inputs before generating a recommendation. If any value was missing, outside the allowable range, or internally inconsistent, AnemiaCare HD did not produce a therapeutic output and instead issued a standardized request for clarification or clinician review. This rule-based input validation ensured that deterministic behavior was preserved and prevented unsafe dosing logic from being triggered by erroneous data.

### Protocol encoding in simulation

#### ESA dose adjustment

ESA recommendations were determined by Hb level and rate of Hb change. Rules included:

- Hb < 9.0 g/dL: ESA increases for gradual Hb declines; provider notification for rapid drops (>0.5 g/dL/week); discontinuation with nephrology review if Hb rose >1.0 g/dL/week.
- Hb 9.0–10.7 g/dL: small ESA increases for slow declines; ESA holds for rises ≥0.6 g/dL/week.
- Hb 10.8–12.0 g/dL: ESA reductions or holds; discontinuation if Hb exceeded 12.5 g/dL.
- Safety rules: adjustments were limited to once every 2 weeks, and dose changes followed exact increments (e.g., 10 mcg).

Temporary ESA holds were required if Hb rose >1.0 g/dL in any 2-week period, with reinitiation at a 25–50% reduced dose once Hb returned to target.

#### Intravenous iron therapy

Iron therapy rules required simultaneous assessment of TSAT and ferritin:

- TSAT < 20% and ferritin < 200 ng/mL: IV iron loading (200 mg × 5 treatments).
- TSAT 20–29% and ferritin 200–800 ng/mL: 100 mg IV iron weekly.
- Ferritin 801–1,200 ng/mL with TSAT 20–35%: 100 mg IV iron every 4 weeks, discontinued if TSAT > 35%.
- Ferritin > 1,200 ng/mL: immediate discontinuation of IV iron.

Cumulative iron exposure was tracked throughout, and IV iron was automatically withheld if ferritin exceeded 1,200 ng/mL.

Within the simulation environment, AnemiaCare HD maintained an internal record of cumulative iron administration and regimen status (e.g., initiation and completion of loading courses), allowing the system to apply protocol rules governing iron discontinuation and withholding when ferritin exceeded defined thresholds. Because the institutional protocol bases iron decisions primarily on ferritin and TSAT rather than the timing of the most recent iron dose, recent iron administration was accounted for indirectly through iron indices and cumulative tracking rather than as a separate input variable.

### Prompt refinement phases

**Figure 1:**
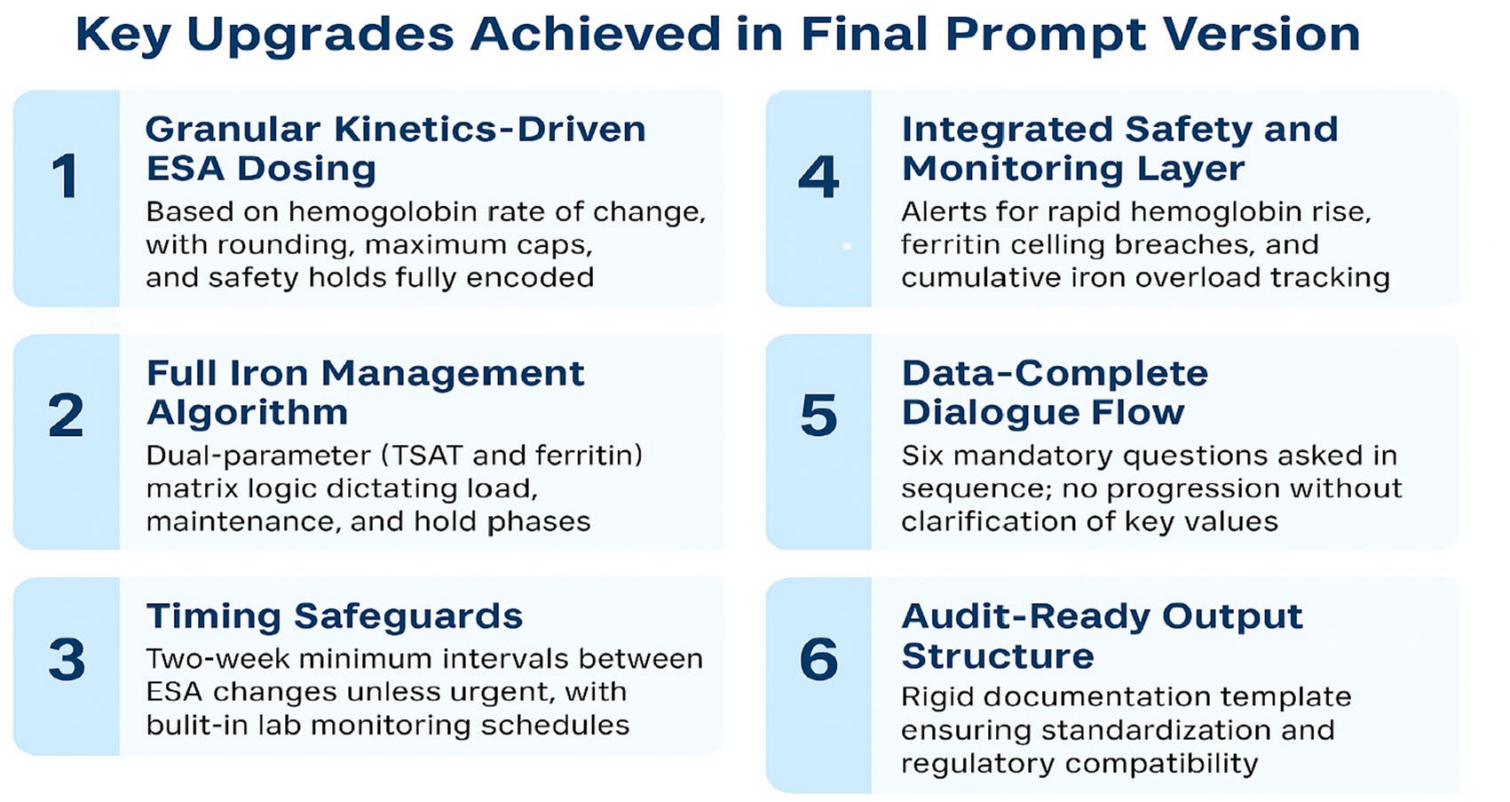
Key upgrades implemented in the deterministic version of AnemiaCare HD.

- Phase 1 (baseline): a loosely defined natural language prompt was applied to 300 simulated cases. The model produced free-text recommendations, which were then evaluated against institutional protocol rules.
- Phase 2 (deterministic): The prompt was redesigned with six major upgrades (Figure 1):

1. Granular kinetics-driven ESA dosing
2. Full iron management algorithm
3. Timing safeguards
4. Integrated safety and monitoring layer
5. Data-complete dialogue flow
6. Audit-ready output structure

This refinement created a deterministic system that generated reproducible, protocol-adherent outputs in all simulated scenarios (Table 1).

### Output review and classification

All outputs were independently reviewed by two board-certified nephrologists with expertise in dialysis anemia management. Two board-certified nephrologists independently reviewed all outputs while blinded to phase assignment. Output files did not include phase identifiers. Although blinding was maintained procedurally, the structured format of Phase II outputs may have allowed reviewers to infer phase membership, which represents a potential limitation. Each recommendation was classified as:

1. Protocol-adherent—matched institutional guidelines exactly,
2. Protocol deviation—incorrect ESA or iron recommendation,
3. Unsafe recommendation—potentially harmful (e.g., iron dosing with ferritin > 1,200 ng/mL),
4. Timing violation—adjustments recommended sooner than 2 weeks after a prior change.

**Figure 2:**
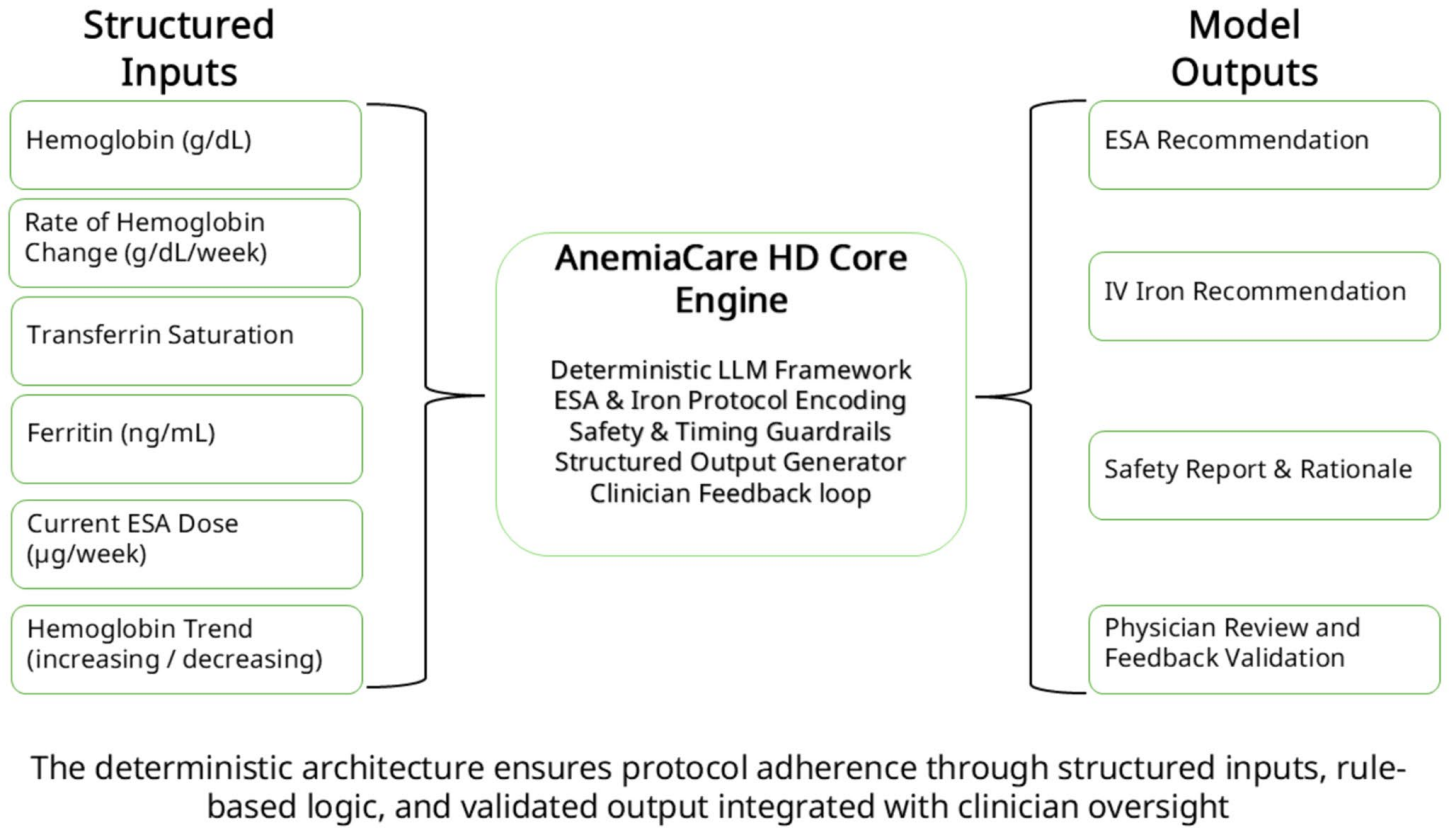
Deterministic LLM architecture of AnemiaCare HD. Structured clinical inputs are processed by a protocol-encoded deterministic LLM that incorporates ESA and IV iron dosing logic, safety guardrails, and feedback validation. The framework outputs protocol-adherent therapeutic recommendations, safety rationale, and clinician-reviewed validation steps, ensuring transparent, reproducible, and rule-based decision support.

Discrepancies between reviewers were resolved by consensus to ensure classification accuracy (Figure 2).

### Outcomes and statistical analysis

The primary outcome was protocol adherence, defined as the proportion of simulated cases with recommendations exactly matching institutional anemia management rules. Protocol adherence was defined as an exact match between the model-generated recommendation and the institutional anemia protocol, including ESA dose adjustment, iron therapy decision, and timing safeguards. Recommendations that were clinically reasonable but deviated in dose magnitude or timing were classified as protocol deviations rather than partially adherent. Secondary outcomes included the frequency of protocol deviations, unsafe recommendations, and timing violations.

Adherence rates were compared between phases using a two-sided Fisher’s exact test, which was selected due to the categorical nature of the data and the presence of small sample sizes and zero cell counts in several outcome categories, making it the most appropriate statistical approach for proportional comparisons in this setting. A two-sided *p*-value <0.05 was considered statistically significant.

This study used exclusively simulated scenarios derived from institutional anemia management protocols and did not involve human participants or identifiable patient data. Therefore, Institutional Review Board review was not required. The work was conducted in accordance with institutional and international ethical guidelines for research that does not involve human subjects. The final, interactive, optimized build of AnemiaCare HD used in this study is publicly accessible^1^ to support full transparency, reproducibility, and independent evaluation.

## Results

In Phase 1, when evaluated with a loosely structured prompt, AnemiaCare HD produced variable outputs. Out of 300 simulated cases, only 96 (32%) were fully protocol-adherent. The majority showed errors that reflected the limitations of free-text prompting. The most common problem was incorrect ESA titration, where recommended dose changes did not align with protocol thresholds. These errors risked both under-treatment and overshooting of Hb levels.

Unsafe recommendations were also common. In several cases, the model advised continuing ESA despite Hb levels above 12.5 g/dL, where discontinuation is required, or suggested IV iron for patients with ferritin greater than 1,200 ng/mL, raising concern for iron overload. Timing violations were also identified, with ESA adjustments sometimes recommended earlier than the two-week minimum. Such premature changes could worsen Hb cycling and add further instability to anemia control. Taken together, these findings highlighted the risks of applying a flexible, general-purpose prompt without strict safeguards.

In Phase 2, after deterministic rules and safety checks were embedded, performance improved dramatically. All 300 simulated scenarios were managed in full compliance with institutional protocols, corresponding to 100% adherence. Every output provided a structured recommendation that included ESA and iron dosing, a rationale linked to protocol thresholds, and built-in safety notes.

### Example input–output pair

#### Inputs

Hb 8.7 g/dL, declining 0.3 g/dL/week, TSAT 18%, ferritin 150 ng/mL, ESA 40 mcg/week.

#### Deterministic output

Increase ESA by +10 mcg and initiate IV iron loading (200 mg × 5).

#### Safety annotation

‘Monitor for rapid Hb drop; reassess ferritin after completion of loading course.’ This example illustrates how the model applies protocol rules and generates structured safety guidance within the constrained template.

All error categories observed in Phase 1 were eliminated. ESA adjustments followed protocol-defined increments and intervals without deviations. Unsafe recommendations were avoided entirely: ESA was consistently withheld when Hb exceeded the limit, and iron was stopped when ferritin was too high. Safety alerts for rapid Hb rise and cumulative iron exposure triggered reliably, ensuring proper handling of edge cases.

The improvement from Phase 1 to Phase 2 was highly significant (32% vs. 100%, *p* < 0.001, Fisher’s exact test). The transition from Phase 1 to Phase 2 marked more than a numerical improvement. It fundamentally changed the system from producing variable, sometimes hazardous outputs to generating reliable, reproducible, and clinically safe recommendations. By encoding explicit ESA kinetics, dual-parameter iron algorithms, timing safeguards, and mandatory safety checks, AnemiaCare HD demonstrated complete fidelity to a complex anemia management protocol—something that general-purpose LLMs have not achieved.

Inter-rater agreement for classification was high (Cohen’s *κ* = 0.92), indicating strong consistency between reviewers in identifying protocol adherence, deviations, unsafe recommendations, and timing violations. Discrepancies were resolved by consensus.

## Discussion

This study demonstrates that a deterministic large language model can achieve complete fidelity to a complex institutional anemia protocol in simulated hemodialysis scenarios. Unlike general-purpose systems, AnemiaCare HD was intentionally designed to merge clinical precision with automation, integrating ESA dosing, IV iron recommendations, and safety safeguards in alignment with Hb trends. To our knowledge, this represents the first LLM framework to deliver an end-to-end, protocol-based approach to anemia management in dialysis.

In Phase 1, loosely defined prompting produced variable and at times unsafe outputs, highlighting the inherent risks of using unconstrained, general-purpose LLMs for medical decision-making. In contrast, after embedding explicit ESA and iron algorithms, timing safeguards, and proactive safety checks in Phase 2, AnemiaCare HD achieved complete adherence, providing consistent, transparent, and reproducible recommendations. This deterministic structure minimizes hallucination risks by restricting model behavior to fully rule-bound clinical pathways, ensuring that outputs cannot deviate outside protocol logic. This aligns with current FDA “Good Machine Learning Practice” (GMLP) principles (Pollard et al., 2022), which emphasize reproducibility, explainability, and traceability as essential characteristics for clinical AI systems. This direct comparison underscores how deterministic prompt engineering can transform a model from generating plausible but unreliable suggestions into one that provides stable, clinically actionable guidance. While the deterministic rule encoding in the present study could theoretically be implemented using a classical rule engine, the use of an LLM provides several additional functions that are not easily achieved with traditional systems. These include the ability to generate structured, clinician-facing explanations that mirror real-world documentation, support natural-language interaction to ensure data completeness and reduce input ambiguity, and produce audit-ready narrative outputs that facilitate transparency and regulatory review. Furthermore, the LLM architecture allows scalable extension to additional clinical domains without requiring complete system reprogramming, offering a flexible platform for future cross-domain reasoning once appropriately validated. In this proof-of-concept phase, the LLM therefore serves as both a deterministic inference mechanism and a communication layer, enabling transparent justification of recommendations while maintaining strict rule adherence. This explainability and traceability component is increasingly emphasized in regulatory guidance for clinical AI systems, where human-understandable justification is required for safe deployment.

The decision to restrict model inputs to a single decision time point reflects the structure of the underlying institutional protocol, which bases ESA and iron adjustments on the most recent laboratory values and dosing history. This design enabled focused evaluation of deterministic rule enforcement and safety behavior without the additional complexity introduced by longitudinal response modeling. As such, the current framework does not attempt to predict future hemoglobin trajectories or account for patient-specific variability in ESA responsiveness, which require time-series data and physiologic modeling to capture accurately.

We acknowledge that the current framework does not incorporate the timing of recent iron administration as a user-provided input. Iron exposure was instead represented through cumulative dosing logic and protocol-based thresholds using ferritin and TSAT. While this approach aligns with the structure of the institutional anemia protocol used for simulation, it does not capture the full physiologic dynamics of iron handling. Future iterations will incorporate time-resolved iron exposure and dosing schedules to support more physiologic modeling and predictive applications. In addition, this work should be interpreted as a method-development and process-validation study aimed at determining whether deterministic prompt engineering can fully eliminate unsafe variability in a controlled simulation environment.

The framework was intentionally not designed to optimize hemoglobin trajectories or demonstrate clinical effectiveness, but rather to establish whether an LLM-based system could reliably enforce complex dosing and safety rules without deviation—a foundational requirement before real-world deployment. As such, the present findings demonstrate technical feasibility and protocol fidelity in a simulated setting, but they do not yet address clinical effectiveness, workflow integration, or patient outcomes. Because adherence categories were rule-based, automated validation could have been performed, and the reliance on nephrologist review added limited incremental expertise. Additionally, the study did not assess longitudinal hemoglobin outcomes or treatment efficiency through retrospective simulation, representing an important limitation. Accordingly, the findings should not be interpreted as evidence that the model improves clinical decision-making or patient outcomes. Real-world implementation will require addressing several practical barriers, including integration within electronic health-record workflows, reliable extraction of structured laboratory and dosing data, clinician acceptance, and ongoing safety monitoring. Regulatory requirements for auditability and update governance will also need to be met to ensure safe deployment in clinical environments.

Over the past several decades, various AI and machine learning techniques, including fuzzy logic, support vector machines, Bayesian networks, and reinforcement learning, have been applied to optimize anemia management in ESKD (Escandell-Montero et al., 2014; Gaweda et al., 2008; Barbieri et al., 2015; Yun et al., 2021; Bellazzi, 1993; Gaweda et al., 2003; Martínez-Martínez et al., 2014). These models demonstrated promise in theoretical work, but their clinical translation has often been constrained by the difficulty of modeling the longitudinal, nonlinear dynamics of Hb and by reliance on narrow input variables that overlook the complexity of dialysis care. However, the ACM represents a significant exception. ACM has been integrated into a dialysis-centered electronic medical record system and deployed internationally, with documented improvements in hemoglobin control, ESA utilization efficiency, and hospitalization rates (Garbelli et al., 2024a,b; Gandjour et al., 2025). These outcomes demonstrate that algorithmic anemia management can achieve meaningful clinical translation when implemented within a structured and well-controlled environment. The limitation we intended to highlight pertains not to deployment feasibility but rather to the challenges many existing systems face with transparency, protocol-specific reproducibility, and interpretability. Most prior models operate as predictive or optimization engines with limited visibility into the dosing rationale, which may hinder clinician trust and regulatory acceptability. In contrast, the deterministic framework developed in the present study was designed to provide fully traceable, rule-based reasoning with explicit safety guardrails, offering a complementary pathway toward responsible clinical decision support integration. AnemiaCare HD addresses these shortcomings by incorporating both ESA kinetics and iron-metabolism dynamics into a deterministic framework, producing recommendations that are accurate, interpretable, and fully protocol-adherent.

The importance of integrating ESA kinetics into AI-based models has been previously demonstrated by McCarthy et al., who used a highly parameterized physiologic model of erythropoiesis to predict ESA response. That model succeeded in maintaining Hb levels within the target range while reducing ESA utilization, underscoring the value of physiologic modeling for individualized dosing (McCarthy et al., 2014). Similarly, AnemiaCare HD builds on this concept by translating established physiologic principles into a rule-driven, transparent LLM framework suitable for bedside implementation. Furthermore, regarding therapy, incorporating iron-metabolism dynamics, based on TSAT, ferritin, and cumulative iron exposure, has also been shown to enhance predictive accuracy in AI-driven anemia management (Inoue et al., 2025).

Hb cycling remains one of the most persistent challenges in anemia management, driven by variable ESA responsiveness and overly frequent dose modifications (Jörg et al., 2023; Collins et al., 2005; Thanakitcharu and Jirajan, 2016). By enforcing strict timing rules and embedding dual-parameter iron algorithms, AnemiaCare HD effectively prevented recommendations that would exacerbate this variability. This observation aligns with findings from Kang et al. (2024), who demonstrated that predictive accuracy in anemia management improves when models use a limited set of clinically meaningful variables, defined by expert knowledge, rather than relying on large, indiscriminate datasets.

The significance of these findings is twofold. First, deterministic prompt engineering can faithfully encode complex clinical rules into reproducible, transparent outputs, an essential foundation for building trust in AI-based clinical decision support. Second, the complete elimination of protocol deviations, including inappropriate ESA use above target Hb thresholds, unsafe iron dosing, and premature adjustments, demonstrates how rule-based design can directly overcome the safety barriers that currently limit LLM use in medicine.

Together, these insights suggest that deterministic LLM frameworks guided by essential, physiology-based variables may help stabilize Hb levels, improve safety, and reduce treatment variability in dialysis care. While the present findings demonstrate that deterministic prompt engineering can fully eliminate protocol deviations in a controlled simulation environment, this framework does not yet address the broader clinical challenges of renal anemia management, including heterogeneous ESA responsiveness, operational constraints, and longitudinal Hb variability. Established ML-based anemia control systems, such as ACM (Garbelli et al., 2024a,b; Gandjour et al., 2025) and physiologic response models, focus on prediction and dose optimization and have demonstrated improvements in hemoglobin stability and treatment efficiency. In contrast, AnemiaCare HD was intentionally designed to prioritize transparency, traceability, and rule fidelity, addressing key barriers to clinical adoption and regulatory acceptance. As such, the current framework should be viewed as a foundational safety layer that could complement predictive or optimization-oriented approaches rather than replace them. Future work will compare deterministic LLM performance with established ML-based systems and evaluate whether integrating physiologic modeling or predictive elements can enhance clinical effectiveness.

There are limitations to acknowledge. All test scenarios were simulated rather than derived from real-world patients, so external validation in clinical settings remains essential. Additionally, the current system was based on a single institutional protocol; thus, its adaptability to other dialysis centers, practice environments, and guideline frameworks warrants further evaluation. In addition, we acknowledge that despite anonymization and randomization procedures, the structured and templated format of Phase II outputs may have allowed reviewers to infer phase assignment based on stylistic cues. Although the high inter-rater agreement suggests consistent application of protocol rules, future evaluations would benefit from masking stylistic features by presenting only standardized dosing outputs or parameter-level recommendations in a uniform format, randomly mixed across conditions.

Despite these limitations, this study provides compelling proof-of-concept evidence that deterministic LLMs can move beyond flexible text generation to deliver reliable, auditable, and protocol-compliant clinical decision support. Future work should focus on integrating AnemiaCare HD into electronic health-record platforms, refining its dosing algorithms using real-world patient data, and conducting prospective validation trials to confirm its safety, scalability, and clinical impact. The deterministic LLM framework developed in AnemiaCare HD demonstrates that reproducibility and safety can coexist within AI-driven clinical decision support. This blueprint can be extended to other guideline-intensive domains such as hypertension, mineral-bone disorder, and transplant immunosuppression, providing a transparent, auditable pathway toward regulatory-grade AI deployment.

A translational gap remains between deterministic performance in simulated cases and meaningful clinical impact. Real-world anemia management involves patient heterogeneity, comorbidities, provider preferences, and operational constraints that cannot be fully captured in simulated protocols. Future work will therefore require multi-center external validation, prospective evaluation embedded within dialysis workflows, and assessment of clinician acceptance, usability, and safety monitoring mechanisms. These steps will be essential to determine whether deterministic LLM systems can enhance clinical decision-making, reduce hemoglobin cycling, or improve treatment efficiency in practice.

## Conclusion

AnemiaCare HD demonstrates that deterministic LLMs can achieve full adherence to complex anemia management protocols by embedding explicit clinical rules, safety safeguards, and physiologic logic. This proof-of-concept highlights their potential to deliver reliable, interpretable, and protocol-compliant decision support in hemodialysis anemia care. By constraining generative flexibility and mandating protocol-coded reasoning, deterministic LLMs such as AnemiaCare HD offer a safer paradigm that may better satisfy regulatory expectations for AI-driven clinical decision support. Future validation in real-world settings is warranted to confirm safety, scalability, and clinical impact. Successful real-world implementation will depend on workflow integration, external validation across diverse practice settings, and alignment with regulatory expectations for transparency and safety.

## Data Availability

The original contributions presented in the study are included in the article/supplementary material, further inquiries can be directed to the corresponding author.

## Footnotes

1
https://chatgpt.com/g/g-680c1a00c1388191be652b9548a9b732-anemiacare-hd-high-fidelity-version
